# Predation of invasive silver carp by native largemouth bass is size-selective in the Illinois River

**DOI:** 10.1038/s41598-023-43470-7

**Published:** 2023-10-06

**Authors:** Eli G. Lampo, Jon M. Vallazza, Cory A. Anderson, Levi E. Solomon, Richard M. Pendleton, Toby J. Holda, James T. Lamer

**Affiliations:** 1 Illinois Department of Natural Resources, Yorkville, IL USA; 2 United States Geological Survey, La Crosse, WI USA; 3 United States Forest Service, Wilmington, IL USA; 4 Illinois Natural History Survey, University of Illinois Urbana-Champaign, Havana, IL USA; 5 Department of Natural Resources, Cornell University, Albany, NY, USA

**Keywords:** Ecology, Behavioural ecology, Community ecology, Conservation biology, Freshwater ecology, Riparian ecology

## Abstract

Silver carp (*Hypophthalmichthys molitrix*) are a nonnative, planktivorous, and highly invasive species of cyprinid located throughout the Mississippi River Basin. Although they co-occur with largemouth bass (*Micropterus nigricans*), an abundant native predatory fish, their predator–prey relationship is poorly understood. This potential relationship warrants investigation as largemouth bass are large-gaped predators capable of exhibiting top-down control on planktivorous fishes. The objectives of this study were to determine if largemouth bass consume juvenile silver carp, and if there was a relationship between length of largemouth bass and length of silver carp consumed. Largemouth bass were collected from the La Grange Pool of the Illinois River using 60 Hz-pulsed DC electrofishing and their diets were analyzed (n = 389, total length = 70–578 mm). Evidence of silver carp was present in 18% of diets of largemouth bass that consumed fish. Lengths of consumed silver carp were estimated from the dimensions of their recovered chewing pads or pharyngeal teeth in the stomachs of largemouth bass. A significant relationship between length of largemouth bass and length of silver carp consumed (*p* < 0.001, F = 34.63, r^2^ = 0.61) was observed. Estimated total lengths of silver carp were 34–101 mm and were recovered from diets of largemouth bass that were 94–262 mm total length. These results indicate enhancement of native largemouth bass populations is unlikely to substantially reduce silver carp populations in the Illinois River or in other waterways where these species co-occur.

## Introduction

Invasive species are a problem for resource managers across a wide variety of ecosystems. Native predator enhancement as a tool to reduce invasive species is an attractive management option and has been considered in aquatic systems^1^. Where effective, this strategy would allow managers to achieve two objectives at once: (1) the reduction of invasive species populations, and (2) the enhancement of native species populations. Enhancement of populations of *Brycon orbignyanus*, a characid native to the Parana River Basin, was shown to offer some control to invasive juvenile fishes such as the blue peacock bass (*Cichla* spp.) and channel catfish (*Ictalurus punctatus*) in controlled studies^2^. Similarly, largemouth bass *Micropterus nigricans* (formerly: *M. salmoides*^3^) have also been considered as a viable species to control nuisance golden shiner (*Notemigonus crysoleucas*) populations in Washington state^4^. Of course, if the native predator population enhanced is a sportfish, the strategy would achieve a third management objective: the enhancement of recreational opportunities. However, native predator population enhancements are met with variable success predicated on the native predator being able to consume sufficient numbers of the invasive prey at a life stage where the reduction in numbers will affect its long-term population abundance.

One behavior that could hinder the success of a native predator population enhancement endeavor is size-selective predation behavior of the native species. Size-selective feeding is likely a result of optimal foraging strategies or gape limitations. Optimal foraging theory was first proposed for bluegill (*Lepomis macrochirus*)^5^ and suggests that predators adapt feeding behavior to maximize net-energy gain based on prey abundance, time spent foraging^6^, and handling time^5^. Handling time is likely affected by gape and prey size. Fishes, more than other vertebrate taxa, tend to corroborate optimal foraging theory^7^. Whatever its cause, size-selective feeding behavior has been demonstrated in several predatory fishes native to North America, including northern pike (*Esox lucius*)^8^, coho salmon (*Oncorhynchus kisutch*)^9^, and largemouth bass^6^. Such behavior could result in a dynamic where the size structures of a predator population and a prey population were mismatched^10^. Such a ‘mismatch’ occurred for largemouth bass feeding on bluegill when bluegill populations were fast-growing^11^.

Silver carp (*Hypophthalmichthys molitrix*) are an invasive species found throughout much of the Mississippi River Basin^12,13^. Rapid growth rates, high fecundity^13^, and their tendency to invade^14^ often perturb aquatic food webs^15–18^. Their rapid growth potential allows them to quickly outgrow predation risk from native predators^14^. Therefore, understanding which native predators consume silver carp and at what frequency, and determining the sizes of silver carp that native predators are capable of eating would be important considerations in the implementation of native predator enhancement in the management of invasive silver carp. Blue catfish (*Ictalurus furcatus*) are known to consume adult silver carp^19^. In addition, several predators native to Midwestern rivers, including shortnose gar (*Lepisosteus platostomus*), white bass (*Morone chrysops*), black crappie (*P*o*moxis nigromaculatus*), channel catfish, smallmouth bass (*Micropterus dolomieu*)*,* largemouth bass, white crappie (*Pomoxis annularis*)*,* yellow perch (*Perca flavescens*)*,* flathead catfish (*Pylodictis olivaris*), freshwater drum (*Aplodinotus grunniens*), and yellow bass (*Morone mississippiensis*)*,* are known to consume young of the year (YOY) silver carp^20,21^. However, information on the frequency of consumption and size limitations of these native predators when consuming small silver carp is generally lacking.

**Figure 1:**
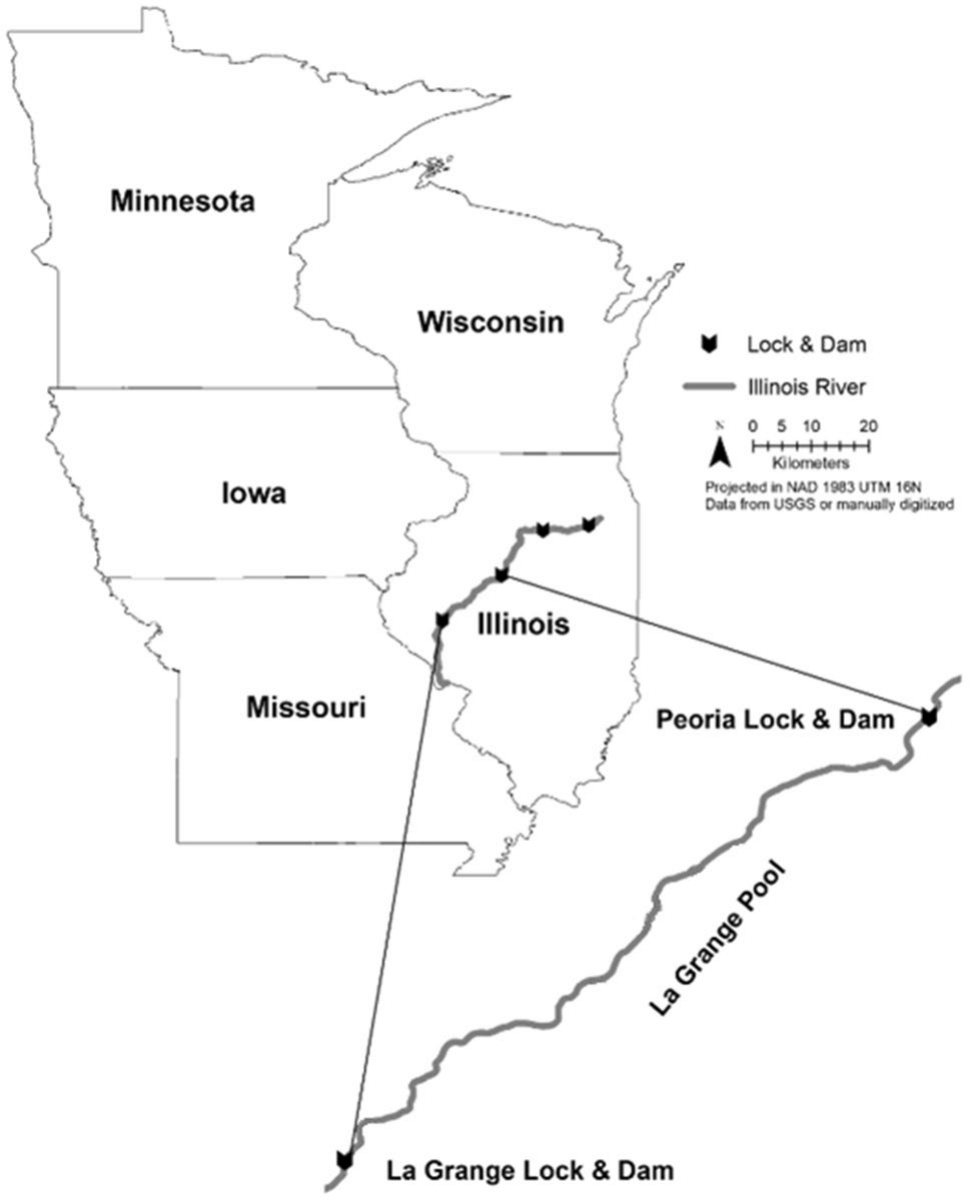
Map of the La Grange Pool of the Illinois River where targeted sampling for largemouth bass was conducted in backwaters, side channels, and tributaries of the river. This map was created using ArcGIS software, version 10.7.1, https://www.esri.com/en-us/arcgis/products/arcgis-desktop/overview.

Largemouth bass are native to the Mississippi River Basin, and are piscivores in lake and river ecosystems^22,23^, where they often consume large numbers of juvenile and YOY planktivorous fish (e.g., gizzard shad, *Dorosoma cepedianum*)^24^. Largemouth bass can consume enough of their prey to elicit a top-down ecological effect on freshwater systems when those prey are abundant and easily-consumed planktivorous fishes^25^. Largemouth bass are one of the most abundant top predators in large Midwestern rivers where silver carp are invading^26^. In addition, largemouth bass are a popular sportfish in U.S. waters^27^. For these reasons, largemouth bass population enhancement could be a very attractive strategy if proven effective for reducing invasive silver carp populations. Largemouth bass have demonstrated selective feeding strategies for prey species other than silver carp based on prey handling time and size^28,29^. Specifically, they have been shown to consume prey with total lengths of 30–50% of their own length^29–32^. Given the lack of information regarding largemouth bass predation on juvenile silver carp, we examined the occurrence and potential size-selection of largemouth bass predation on juvenile silver carp. The study was conducted during 2015 in the La Grange Pool of the Illinois River (Fig. 1) where largemouth bass are abundant and silver carp frequently have strong year classes^26^. We predicted that largemouth bass in the La Grange Pool of the Illinois River would consume silver carp of similar relative size and at similar frequencies as observed previously with other species.

## Methods

### Largemouth bass and silver carp collection

We retained all largemouth bass and silver carp collected during targeted, variable-effort, electrofishing (60 Hz, pulsed-DC) surveys by Western Illinois University (WIU) field crews during June 2015–November 2015. Largemouth bass > 70 mm (n = 389) were retained for diet analysis and silver carp < 375 mm (n = 494) were retained to establish relationships between chewing pad/pharyngeal teeth dimensions and total silver carp length (refer to Lampo et al.^33^). Based on studies indicating that largemouth bass consume prey only 50% of their length or less^29–31^, largemouth bass < 70 mm would only be consuming YOY silver carp. Therefore, we did not collect any largemouth bass smaller than 70 mm for diet studies because the predation of YOY silver carp by largemouth bass was already being studied in the La Grange Pool of the Illinois River^20^. Electrofishing specifications were standardized according to the Upper Mississippi River Restoration Program Long Term Resource Monitoring (LTRM) element protocol^34^, and then adapted to a species-targeted design (variable effort for largemouth bass and silver carp; variable electrofishing settings for silver carp capture). Electrofishing by WIU was conducted at backwater, side channel, and tributary shorelines (Table 1) of the La Grange Pool of the Illinois River (Fig. 1) where we expected to find largemouth bass and juvenile silver carp. Length (mm) and weight (g) were recorded for all largemouth bass and silver carp retained prior to euthanasia. Institutional Animal Care and Use Committee (IACUC) review and approval were documented before sample collection (IACUC Protocol 16-10r at WIU), and guidelines were followed to ensure humane euthanasia of retained fish. In addition, the ARRIVE reporting guidelines^35^ were followed in the preparation of this manuscript. Fish were packed on wet ice in the field to slow digestion and avoid decomposition and frozen whole at − 10 °C upon return to the laboratory. Largemouth bass were later thawed, and stomachs were removed and placed in 70% ethanol to facilitate diet analysis. Silver carp were later thawed, and hard structures (otoliths/pharyngeal teeth) were removed.

**Table 1:** Locations for sampling of largemouth bass collection for diet analyses by Western Illinois University field crew in the La Grange Pool of the Illinois River (see Fig. 1).

Site Name	Coordinates	Site Description
Meyers Ditch	40.390303°, − 89.985863°	Backwater
Lily Lake	39.978252°, − 90.509580°	Backwater
Coal Ditch	40.310957°, − 90.063856°	Backwater
Rice Lake	40.476498°, − 89.946731°	Backwater
Quiver Lake	40.340299°, − 90.040138°	Backwater
Clear Lake	40.405017°, − 89.9485046°	Backwater
Bath Chute	40.220127°, − 90.137372°	Side Channel
Spoon River	40.307586°, − 90.070919°	Tributary
Browning Side Channel	40.096783°, − 90.386460°	Side Channel
Panther Slough	40.129954°, − 90.341930°	Backwater
La Moine River	39.985868°, − 90.515295°	Tributary
Beardstown Marina Cove	40.024570°, − 90.427898°	Backwater
Treadway Lake	40.081556°, − 90.381247°	Backwater

### Largemouth bass diet analysis

Largemouth bass stomachs were dissected, and diet items were identified to the lowest taxonomic level (species for fish, if possible) and enumerated. Prey fish were identified by external morphology (if not digested beyond recognition), hard structures, or genetic analysis if fish were unidentifiable. To ensure there was a 1:1 correspondence between fish and tissue, only tissue connected to unknown fish vertebrae were removed for genetic analysis. Digestion made it difficult to identify silver carp by conventional anatomy and most could only be identified by their unique chewing pad and pharyngeal teeth structures^20,33^. The chewing pad is a keratinized structure located at the posterior end of the palatal organ^33,36^ and these are identical for both silver and bighead carp. As no bighead carp were collected in 2015 in our sampling, nor Illinois River Biological Station (IRBS) sampling, nor genetic analysis, we assumed all chewing pads came from silver carp. Grass carp and other cyprinids have pharyngeal teeth, but they do not resemble those collected from silver carp. The pharyngeal arch refers to the 5th modified branchial arch, which contains the pharyngeal teeth^33,37^. All empty largemouth bass diets were omitted from this study. A frequency of occurrence analysis (proportion of fish that contained a given food type)^38^ of major diet items was conducted on largemouth bass diets containing identifiable fish (n = 169 of 389, not including fish with empty stomachs, just macroinvertebrates, or just plant matter) to determine which types of prey fish were present in the largemouth bass diets. Prey fish were grouped by families, except for the invasive carp species (silver carp and grass carp *Ctenopharyngodon idella*).

### Genetic analysis protocol

Some diet items were heavily digested, making conventional prey species identification impossible. Genetic analysis was performed to determine prey species of heavily digested diet items. We used the method previously described by Anderson et al.^20^ and Locher et al.^19^ because it was optimized for the suite of prey items in the Illinois and Mississippi Rivers, especially including the invasive silver carp. Briefly, we extracted DNA from unknown tissues collected during diet analysis and determined consensus prey species identification by using barcode primer sequences designed for use in fishes, modified from Ivanova et al.^39^, Leray et al.^40^, Sarri et al.^41^ and De Barba et al.^42^. Consensus identification for each diet content was reached based on repeatability across multiple domains and identity score^19^. Non-fish identifications and highly improbable assignments due to geography or taxa were discarded^19^. Because some fish were identified only to family level using hard structures found during diet analysis, fish identified to species by genetic analysis were then grouped into their respective families for the frequency of occurrence analysis. Methods were carried out in accordance with all relevant guidelines and regulations.

### Ingested silver carp length estimation

Measurements of silver carp chewing pads and pharyngeal teeth recovered from largemouth bass diets were used to estimate silver carp total length according to Lampo et al.^33.^ The primary predictors of silver carp length used were relationships for chewing pad length (from Lampo et al.^33^): (silver carp length = 0.0294 × (chewing pad length) − 8.52) and chewing pad width (silver carp length = 0.0324 × (chewing pad width) − 11.51); however, in the absence of intact chewing pads the dimensions of pharyngeal teeth were used to predictor silver carp length (silver carp length = 0.054 × (PT1_L_) − 9.513). Pharyngeal teeth condition varied between diets (some were cracked or damaged); therefore, when pharyngeal teeth were used to estimate silver carp length, the dimensions used for the pharyngeal teeth regressions and silver carp length estimation were selected based on the strongest predictive relationship (highest R^2^ value) according to Lampo et al.^33^. Estimated lengths of consumed silver carp were regressed against lengths of consuming largemouth bass using R statistical computing software (version 4.2)^43^. In addition, the length of silver carp expected to be targeted by stock, quality, and preferred length bass were predicted from the relationship using Gablehouse’s^44^ largemouth bass stock indices.

### Size structure data

We pooled length data for largemouth bass and silver carp collected for preceding analyses to establish size structures for both species. In addition, to supplement our size structure data from fish we collected for diet analysis, we have included size structure data collected by IRBS for largemouth bass (n = 905) and silver carp (n = 83). The IRBS sample sites were based on a stratified random sampling design, and fish were collected using electrofishing and site-selection specifications specific to the Upper Mississippi River Restoration Program LTRM element^34^. The IRBS data therefore reflect a broader-scale representation of river habitats (a representative mix of main channel, side channel, and backwater) than do the WIU data (primarily backwaters where largemouth bass could be found at higher Catch Per Unit Effort CPUE). The largemouth bass collected from IRBS were not used in diet analysis, and the silver carp collected from IRBS were not used to establish relationships between chewing pad/pharyngeal teeth dimensions and total silver carp length in Lampo et al.^33^.

### Ethical approval

Institutional Animal Care and Use Committee review and approval was documented before sample collection (WIU 16-09).

## Results

### Largemouth bass diet analysis

Diets were examined of 389 WIU-collected largemouth bass (70–578 mm total length), of which 220 had empty stomachs or non-fish diets. A total of 30 largemouth bass examined had consumed silver carp (i.e., 18% of the 169 that consumed identifiable fish prey) based on physical evidence and genetics analyses of diet contents. The most widely consumed prey fish found in largemouth bass diets were Centrarchids, followed by gizzard shad (Table 2). Silver carp were the 3rd most widely consumed prey fish item in largemouth bass diets. Other prey fish consumed included Cyprinids, freshwater drum, catfishes, grass carp, buffalo, mosquitofish *Gambusia affinis*, and white bass (Table 2). Silver carp were only consumed by largemouth bass during the June–August 2015 time period. All of the largemouth bass containing silver carp in their diets were collected from backwater habitats, rather than side channel or tributary habitats (98.5% of the largemouth bass we were able to collect for diet analyses were collected from backwater habitats, with 1.0% percent coming from side channels and 0.5% percent coming from tributaries). Measurable silver carp structures were present in just 20 of the 30 largemouth bass diets that consumed silver carp. Most of those 20 largemouth bass consumed only one silver carp (n = 17, 85% of those that consumed silver carp); however, 2 of the largemouth bass each contained evidence of 2 silver carp (i.e., two sets of chewing pads), and 1 contained evidence of 3 silver carp (i.e., three sets of chewing pads), for a total of 24 silver carp in 20 largemouth bass diets.

**Table 2:** Percent frequency of occurrence (%FO) per prey taxon as observed in the diets of the 169 largemouth bass that consumed identifiable fish.

Prey taxon	Common name	Total %FO
Centrarchidae	Sunfishes	24
Clupeidae	Shads	21
*Hypophthalmichthys*	Silver Carp	18
Cyprinidae	Minnows	14
Scianidae	Freshwater Drum	13
Ictaluridae	Catfishes	8
*Ctenopharyngodon*	Grass Carp	6
Catostomidae	Suckers	3
Poeciliidae	Mosquitofish	2
Moronidae	Temperate Basses	1

Note that Cyprinidae does not include silver carp nor grass carp species.

**Figure 2:**
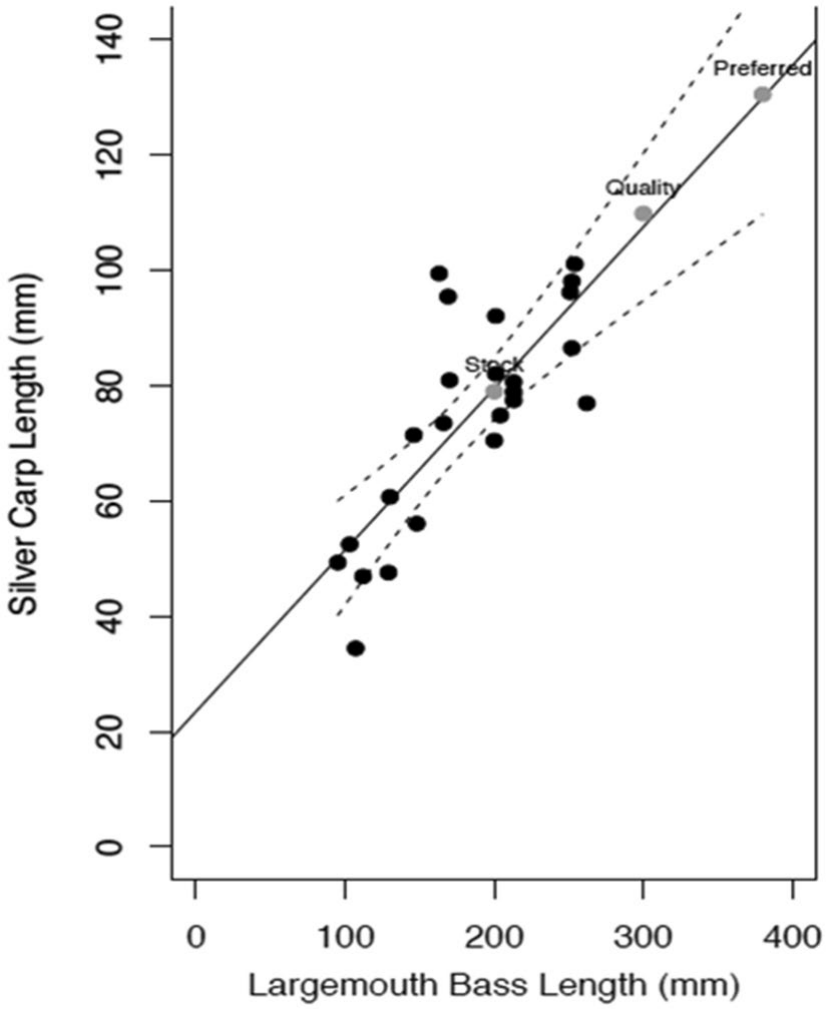
Relationship between largemouth bass total length and the estimated total length of consumed silver carp (95% confidence interval shown with dotted lines) (*p* < 0.001, F = 34.63, r^2^ = 0.61, silver carp length = (0.2802 × largemouth bass length) + 23.502, ± 8.9). Hypothetical estimated total length of silver carp consumed by largemouth bass of minimum sizes for the Gablehouse Stock Index categories^44^ (gray dots) are plotted based on this relationship. All largemouth bass with silver carp in their diets were collected from backwater habitats.

Partial digestion of all the silver carp precluded direct measurement of total length. For the 18 consumed silver carp for which we had chewing pads, we used chewing pads for the length estimation (even if pharyngeal teeth were present). For the remaining six of the consumed silver carp we had to use pharyngeal teeth. Total length estimates of silver carp from hard structures^33^ ranged from 34 to 101 mm. The silver carp consumed were on average about 42% (range of 29–61%) of the total length of the largemouth bass. A positive linear relationship was observed between the size of largemouth bass and silver carp prey (*p* < 0.001, F = 34.63, r^2^ = 0.61, slope = 0.2802 ± 0.05, intercept = 23.504, ± 8.98) (Fig. 2). This indicates that a minimum stock-sized largemouth bass (200 mm) would consume silver carp of 74–84 mm (mean = 79 mm), that a minimum quality-sized (300 mm) largemouth bass would consume a silver carp of 94–120 mm (mean = 107 mm), and that a minimum preferred-sized largemouth bass (380 mm) would consume a silver carp of 110–148 mm (mean = 129 mm) (Fig. 2).

### Silver carp size structure in the La Grange Pool, Illinois River in 2015

**Figure 3:**
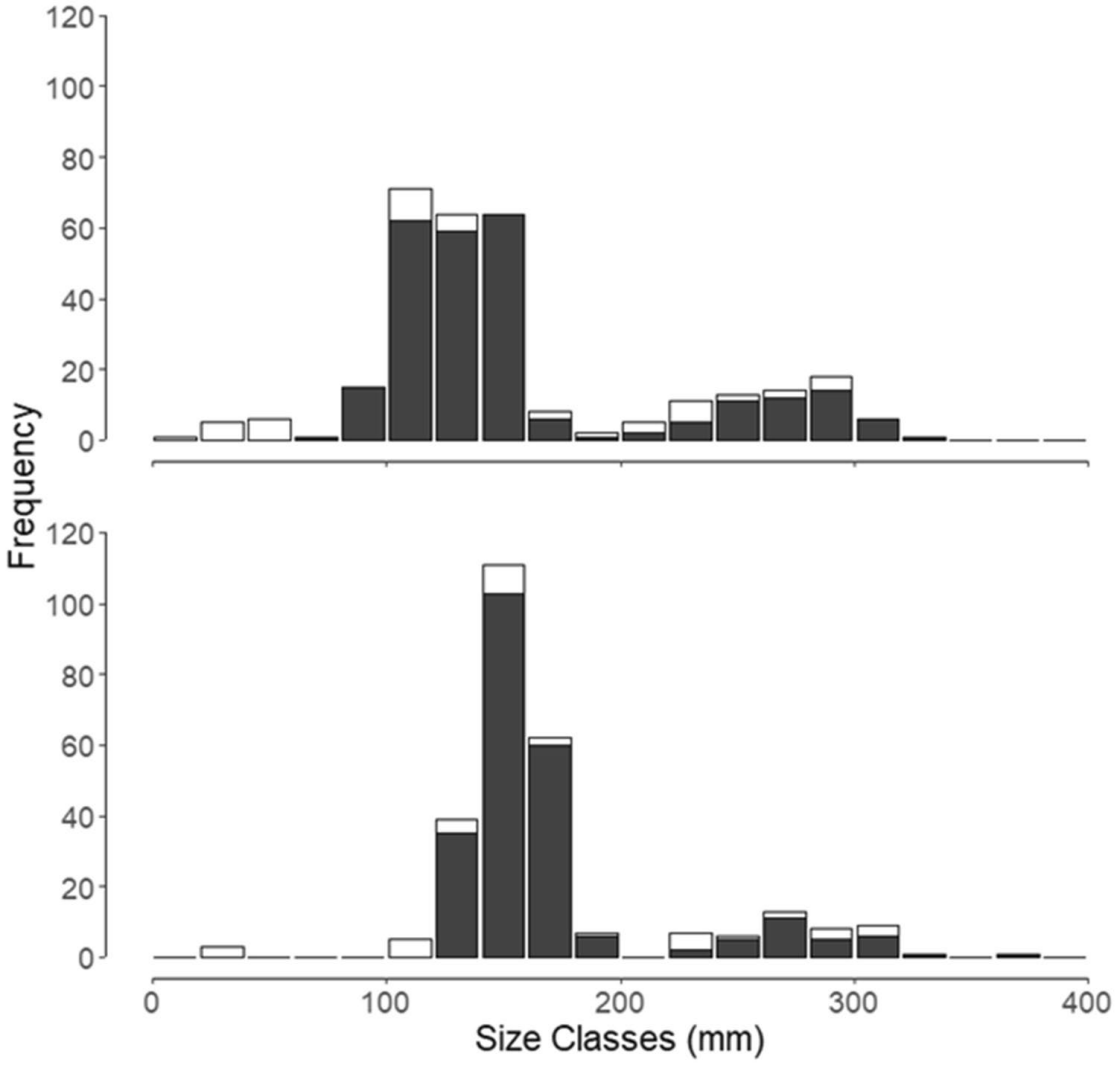
Histograms of size classes of silver carp (n = 577). Top histogram depicts size class of silver carp collected from June through August 2015 (time period where silver carp were consumed by largemouth bass, n = 305). Bottom histogram depicts size classes of silver carp collected during September through November 2015 (time period where no silver carp were consumed by largemouth bass, n = 272). Histograms show total number captured per size class, with amount in white representing number captured by Illinois River Biological Station (IRBS) surveys and amount in dark gray representing number captured by Western Illinois University (WIU) surveys.

A total of 577 silver carp (WIU) = 494; IRBS = 83; 30–373 mm) were collected from June 1–November 9, 2015. We split the length data into two temporally delineated groups based on when silver carp were observed in largemouth bass diets. During June through August 2015, silver carp were observed in largemouth bass diets, whereas no silver carp were observed in largemouth bass diets from September through November 2015. The median length (mm) for silver carp during the months of June–August 2015 (time period where largemouth bass consumed silver carp) was 137 mm and the range was 10–320 mm (Fig. 3). The median length of silver carp during the months of September–November 2015 (time period where no largemouth bass consumed silver carp) was 156 mm with a range of 30–373 mm (Fig. 3). Of particular note, no silver carp were in the size range 40–80 mm during September–November 2015.

### Largemouth bass size structure in the La Grange Pool, Illinois River in 2015

**Figure 4:**
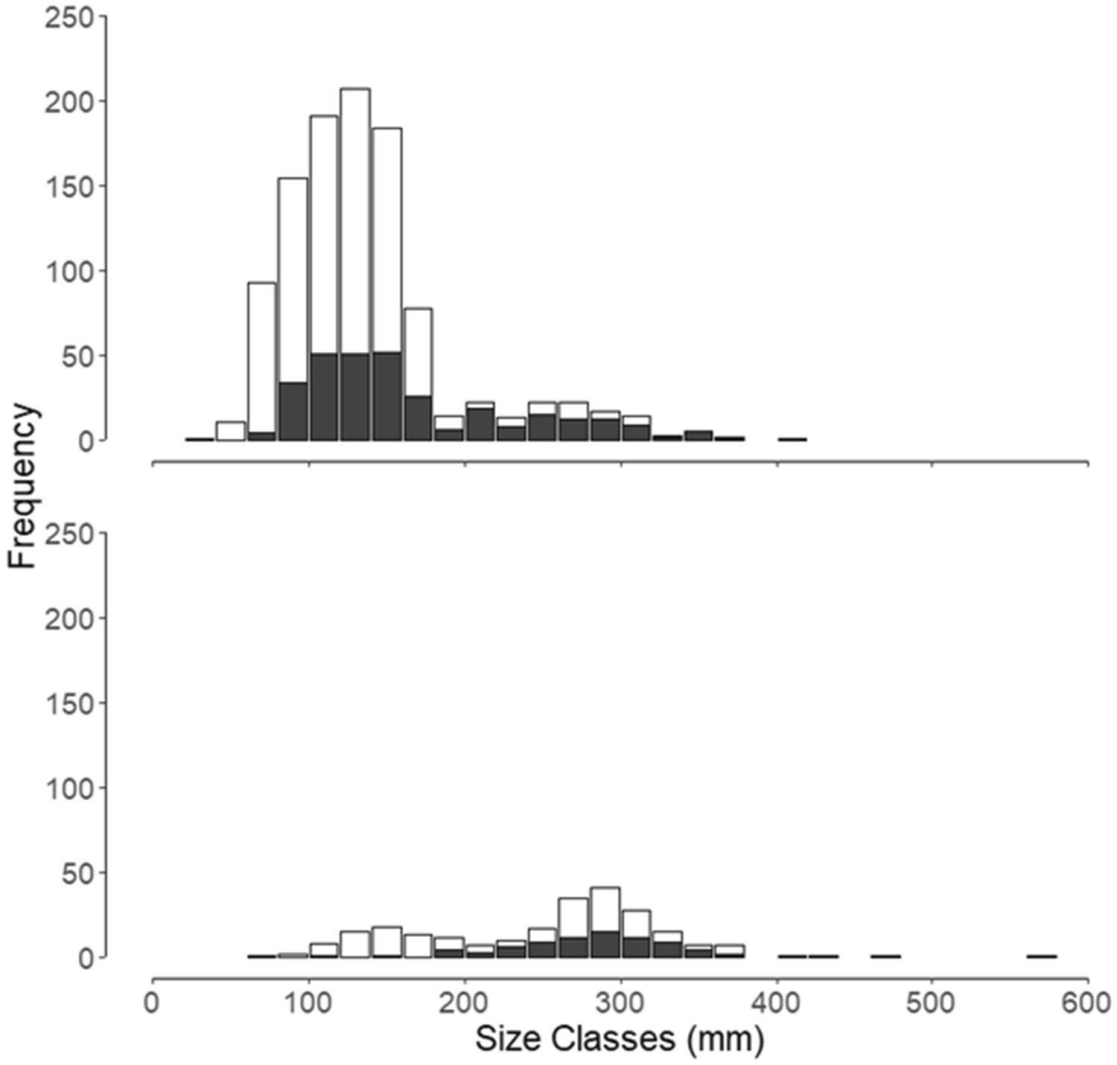
Histograms depicting size classes of largemouth bass collected in the La Grange Pool by Western Illinois University (WIU) and Illinois River Biological Station (IRBS) of the Illinois Natural History Survey during 2015 (n = 1294). Top histogram depicts size class of silver carp collected from June through August 2015 (time period where silver carp were consumed by largemouth bass, n = 1054). Bottom histogram depicts size classes of silver carp collected during September through November 2015 (time period where no silver carp were consumed by largemouth bass, n = 240). Histograms show total number captured per size class, with amount in white representing number captured by IRBS surveys and amount in dark gray representing number captured by WIU surveys.

Collections of largemouth bass by WIU (n = 389, 70–578 mm) and IRBS (n = 905, 30–415 mm) were used to represent the size structure of largemouth bass in La Grange Pool during 2015 (Fig. 4). Using the same temporal periods as silver carp, total length of largemouth bass ranged from 30 to 410 mm during June–August 2015 (median 127 mm). Largemouth bass total length during September–November 2015 ranged from 70 to 578 mm (median 264 mm; Fig. 4). Based on LTRM and WIU electrofishing data in the La Grange Pool in 2015, the predominant sizes of largemouth bass were sub-quality length (< 300 mm, 92.3%), followed by quality length fish (> 300–380 mm, 7.3%), and then preferred length fish (> 380 mm to 510 mm, 0.1%). The LTRM surveys in the La Grange Pool lacked memorable and trophy sized largemouth bass (> 510 mm).

## Discussion

Our study adds to the evidence that largemouth bass do consume silver carp in natural environments, but also adds to the evidence that largemouth bass consume native prey fish more widely than they consume silver carp^20^. However, in our study that focused on juvenile silver carp, silver carp were consumed less widely by largemouth bass than observed in the study that focused on YOY silver carp^20,45^. Silver carp in our study were only the 3rd most-widely consumed prey fish taxon at an 18% frequency of occurrence, behind gizzard shad at 21% and Centrarchids at 24% (but more than more than non-invasive Cyprinids at 14%). In Anderson^45^, silver carp were the second-most widely consumed taxon at 31% frequency of occurrence, behind only gizzard shad at 38%. Gizzard shad have been documented as a key component of largemouth bass diets^24^. Similarly, in both field and laboratory studies that included silver carp and gizzard shad prey, gizzard shad were preferred by largemouth bass^20,21^. Silver carp were actually selected against in the laboratory study^21^. So, it is not unexpected that gizzard shad surpassed silver carp frequency of occurrence in largemouth bass diets both in our study and in Anderson^45^. It is somewhat unexpected that Centrarchids surpassed silver carp frequency of occurrence in our study. Centrarchids were selected against and silver carp were selected for in the field together as reported in Anderson et al.^20^. Centrarchids were also selected against in the laboratory in the presence of bighead carp^21^. However, Centrarchids have, similar to gizzard shad, been documented as a key component of largemouth bass diets^46^.

In addition, although Anderson et al.^20^ documented that largemouth bass consumed YOY silver carp, they were unable to construct prey lengths from digested structures because the relationship^33^ had not been established at the time of their study. Using methods described in Lampo et al.^33^, we were able to present the first evidence to our knowledge of largemouth bass utilizing juvenile silver carp size classes (80–100 mm) as a prey resource in their natural habitat. Further, we were able to show that largemouth bass predation of both YOY and juvenile silver carp was size-dependent. Largemouth bass consumed silver carp at a mean total length of 42% (range of 29–61%) of their own total body length, despite a wide range of juvenile silver carp sizes available. The size of silver carp that we found to be vulnerable to largemouth bass predation falls within the range reported by other studies that indicate largemouth bass typically target prey items 30–60% of their total body length^29–32,47^. Despite a wealth of research on silver carp invasion biology and management, our results are the first evidence to support size selectivity by a native predator when consuming silver carp.

Given the size selective foraging behavior exhibited by largemouth bass feeding on silver carp, the rapid growth of silver carp likely limits the amount of time that they are vulnerable to predation. Although we only observed silver carp in the La Grange Pool measuring 80–100 mm at the end of their first growing season and 110–220 mm after their first year, they can obtain first-year growth as high as 300 mm in populations with low densities^13^. According to the predator to prey size relationship we observed, only largemouth bass 300–630 mm in the La Grange Pool would prey upon the age-1 silver carp we collected. Largemouth bass collected by LTRM in the La Grange Pool in 2015 were about 30–415 mm total length with the majority (94%) being under 300 mm (Fig. 4). Low abundance of quality sized and greater largemouth bass and the predator to prey size relation we observed indicates that silver carp in the La Grange Pool would generally be invulnerable to predation by largemouth bass within their first or second year of life. The window for silver carp from low-density populations (leading edge), where they have reached average lengths of 200–300 mm as young-of-year^13,48^, would outgrow predation even sooner than those in La Grange Pool where densities of silver carp are high.

In our study, silver carp were found in largemouth bass diets during the months of June–August 2015, but not during September–November 2015. Given our evidence for size-selective predation of largemouth bass on silver carp, one potential explanation would be that silver carp were absent or at least insufficiently abundant at optimal foraging sizes for the largemouth bass sampled in WIU collections in the fall. Silver carp size structure data from the La Grange Pool showed a size class shift in 2015, with median silver carp length increasing from 137 mm in June–August to 156 mm in September–November. Perhaps more important was the total absence of silver carp in the 40–80 mm size range, which based on our findings, would be the optimal foraging size (at 40%) for largemouth bass from 100 to 200 mm. Thus, it is possible that the shift in size classes allowed silver carp to escape predation risk from largemouth bass during the late sampling period. However, we acknowledge that silver carp from 80 to 140 mm in the fall would have been optimal foraging size for the largemouth bass from 200 to 350 mm that also were abundant at that time. Thus, other potential explanations include the possibility that largemouth bass and/or silver carp behavior or habitat preferences changed from June–August to September–November in a way that reduced their encounter rate. However, the evidence during June–August for size-selective predation, combined with the size structure shift from June–August to September–November, indicate that optimal size selection likely played a role in the absence of silver carp in largemouth bass diets during September–November of 2015. In Wisconsin lakes, bigger largemouth bass have been shown (over a period of 2 years) to have less optimal feeding and eventually non-feeding on bluegill populations that were faster growing as compared with bluegill populations that were slower growing^11^. Thus, given the high growth rates of silver carp in the Illinois River, the same process would be expected to occur there on even shorter time scales.

Another consideration in the effects of size-selective predation is whether the timing of abundant prey resources such as YOY silver carp overlaps with the presence of the right size of predators such as largemouth bass. Silver carp reproduction and recruitment is highly variable, leading to boom or bust year-class strength depending on the hydrology and other factors^48^. For example, LTRM daytime electrofishing catch-per-unit-effort of silver carp on the La Grange Pool of the Illinois River was 25.16 (± 10.18) per 15 min in 2014 compared to 2.12 (± 0.49) in 2015^26^. Annual fluctuations in river discharge can also influence the number and timing of spawning events and thus the size structure of YOY silver carp within a given year^49^. This is important because prey abundance plays a role in largemouth bass feeding strategies^6^. Specifically, abundances of ideal prey sizes have been linked to a shift to a more fish-based diet in juvenile largemouth bass^47^. Largemouth bass may not target silver carp or respond to their presence the same way as they did in 2015 if silver carp abundance were low or if (as hypothesized for cod feeding on flatfishes off the coast of Scotland^10^) a predator to prey size mismatch were to occur. This variability would need to be considered in assessing the potential role of largemouth bass in helping control silver carp populations.

Better understanding of size-selection by native predators like largemouth bass provides a foundation for determining if native predator enhancement might be useful as a management option for invasive silver carp. If native predators do prove effective in reducing invasive species populations, then managers could consider increasing their numbers through stocking or targeted habitat enhancement for native predators, which may be beneficial for reducing abundance of invasive species and provide increased angling opportunities for these native fish species. In this study we observed a size-selective predation relationship between largemouth bass and silver carp in the La Grange Pool of the Illinois River. Although largemouth bass might have some potential to contribute to the control of invasive silver carp, our results indicate that this potential is very limited. Largemouth bass in the La Grange Pool consumed silver carp, but they consumed other native fishes more widely (also observed by Anderson^45^ and consistent with observations of Sanft et al.^21^). Largemouth bass were also limited in the size range of silver carp that they preyed upon. In addition, based on the size structure of largemouth bass in the La Grange Pool in 2015, silver carp outgrew the size range where most largemouth bass could consume them once the silver carp reached around 150 mm. This indicates largemouth bass in the Illinois River have a limited timeframe in which to consume silver carp before the silver carp grow too large. Even by the fall of 2015, silver carp juveniles and YOY were no longer being consumed by largemouth bass. Whether this was due to the silver carp outgrowing the optimal foraging size of the largemouth bass, or due to a behavioral or environmental factor, the fact remains that largemouth bass no longer consumed silver carp in the fall. By their second spring, the silver carp will have reached lengths^13,14^ at which they are invulnerable to largemouth bass predation (based on extrapolation of the size selective predation behavior of largemouth bass that we measured in this study). The results of this study indicate that although enhancement of largemouth bass and other native predators in the La Grange Pool of the Illinois River could potentially be used to suppress YOY and smaller juvenile silver carp when densities of these size classes are abundant, it is not likely to prove an effective management option.

Enhancement of native predator populations may have possible unintended consequences on non-target species and to food-web dynamics; thus, careful consideration is warranted. The techniques presented here can also serve as a guide for how to explore the vulnerability of silver carp to native predators other than largemouth bass. Other potential predators capable of preying on silver carp are present in the Illinois River, including shortnose gar and white bass at high abundances and flathead catfish, bowfin (*Amia calva*)*,* channel catfish, and sauger (*Sander canadensis*) at lower abundances. Further research regarding predator–prey relationships between silver carp and other native predators may indicate if native predator enhancement can be a viable invasive silver carp management option with a native predator other than largemouth bass.

## Data Availability

The data for this study are published at: Vallazza, J.M., Lampo, E.G., Anderson, C.A., Solomon, L.E., Pendleton, R.M., Holda, T.J., and Lamer, J.T., 2023, Fish sampling data in La Grange Pool of the Illinois River during 2015 to determine largemouth bass and silver carp population size structure and size-selective largemouth bass piscivory: U.S. Geological Survey data release. 10.5066/P9EE52ZX.
